# Treatment of partial thickness hand burn injuries in children with combination of silver foam dressing and zinc-hyaluronic gel: Case reports

**DOI:** 10.1097/MD.0000000000009991

**Published:** 2018-03-30

**Authors:** Gergo Jozsa, Peter Vajda, Andras Garami, Alexandra Csenkey, Zsolt Juhasz

**Affiliations:** a Department of Paediatrics, Surgical Division; b Institute for Translational Medicine, Medical School, University of Pécs, Hungary.

**Keywords:** children, second burn hand injury, silver foam dressing, treatment, Zn-hyaluronic gel

## Abstract

**Rationale:**

Burns is a common type of traumatic injury in childhood. Nowadays, several wound dressings are available to treat the second-degree hand burns conservatively.

**Patient concerns, diagnoses:**

At the authors’ institute, 37 children were treated conservatively with a special dressing at first intervention containing Aquacel Ag foam and Zn-hyaluronic gel to determine their effectiveness on partial thickness hand burns.

**Interventions:**

The dressing was checked on the second day, and removed on the sixth or seventh day (unless it had spontaneously separated).

**Outcomes:**

None of the 37 children treated with this dressing were diagnosed with wound infection. The authors observed the epithelialization of the burned areas on the 6-7th day after primary conservative treatment. The dressing efficiently promotes epithelialization in all cases. Further advantage of Zn-hyaluronic gel is to enhance cell regeneration and inhibits dressing fixation into the wound.

**Lessons:**

Based on the authors’ experience, with this special combination of wound dressing, a gentle, child-friendly, cost-effective treatment and excellent wound healing observed with favourable cosmetic results.

## 1 Introduction

Burn injury in childhood is mainly caused by extreme heat, electricity, chemicals, friction, or radiation. The most frequently affected age-group is the one <5 years of age.^[1]^

The most common cause of hand burn injury in children is the scald from hot water. Mechanism of the injury, duration of the exposure, depth and range of the burns, age, and general well-being of the child are all important influencing factors of the disease.^[2]^ Burns can be classified by the severity of damage to the skin layers (depth of burns) and the affected surface area of the skin (usually expressed as percentage of total body surface area [TBSA]).

The most moderate lesion – called as first-degree burn (redness of the skin, like sunburn) – affects only the superficial skin layers without blisters or a wound. Generally, these superficial burns do not need medical care.

Partial thickness lesion or second-degree burn (blisters covering a red base*)* reaches the deeper skin layers, extending to the whole epidermis and the dermis. They can be divided into 2 further groups:

(1) Second-degree superficial partial burn (II/A or II/1) penetrates into the dermo-epidermal papillary region.^[2]^ Therapy of this degree is mainly conservative. Several conservative methods are known, such as bandaging with cream or solution, which contains silver nitrate, impregnated webs, modern foam, as well as vacuum therapy.
(2) In cases of partial burns, which extend to the deeper layer (II/B or II/2), also the reticular layer of the dermis is damaged. In this case, the appropriate treatment is a surgical intervention to tangentially excise the necrotic skin part (debridement).

In mixed type of second-degree burn (II/A and II/B), in case of an appropriate indication, conservative treatment methods can also be used.

Third-degree burn (widespread thickness with a white, leathery appearance) extends to all layers of the skin and occasionally even further. Bones can be involved in fourth-degree burn (carbonization), the affected area is homogeneously black and charred. These kinds of injuries require complex surgical interventions.

Aquacel Ag (ConvaTec, USA) foam is a hydrofiber dressing which consists of a superficial polyurethane waterproof layer and a multi-layered absorbent surface containing 1.2% ionized silver. The dressing absorbs the wound secretion as the hydrofiber layer transforms into gel, which facilitates wound-humidification, faster healing, and protects against infections.^[3–5]^ However, only a limited number of studies exist in the pediatric population about its effectiveness. The main component of Curiosa gel (Richter, Hungary) is Zinc-hyaluronic acid, which promotes cell regeneration, therefore, it contributes to faster regeneration of the wound.^[6,7]^

Clinical studies with application of either Aquacel Ag foam or Curiosa gel have been conducted and beneficial effects of each treatment have been found in superficial second-degree burns.^[8]^ However, according to our knowledge, no data are available on the effect of the combination of these treatments in the same burn types.

The aim of this study is to present the results obtained in children with superficial and mixed second-degree hand burn injuries treated simultaneously with Zinc-hyaluronic gel combined with a special silver foam dressing.

## 2 Patients and methods

Between January 1, 2014 and January 31, 2017, a prospective clinical study was performed at the Surgical Division, Department of Paediatrics, Medical School, University of Pécs, Hungary. Thirty-seven children with superficial and mixed-type of second-degree hand burns were included in the study in whom the burning injury was treated with Zn-hyaluronic gel combined with Aquacel Ag foam. In nearly 90% of the cases, burn depth was undoubtedly superficial. Aquacel Ag foam dressing with Zn-hyaluronic gel was applied primarily after wound cleaning and blister removal, which included the removal of the vesicles and blisters (i.e., bullectomy which is not equivalent of necrectomy), but not the burned epidermis. In cases, when the burn depth was not clearly assessable (II/1 or II/2) by the primary surgeon, silver nitrate solution was used for 24 hours. On the following day, burn depth was reassessed by a burn specialist (consultant). At primary treatment, wound cleaning and blister removal was carried out under sedation or general anesthesia. When the burn was superficial (II/A), the above described conservative therapy (Aquacel Ag foam dressing with Zn-hyaluronic gel) was applied. In cases, when the burn depth was II/B degree (or deeper), the patients were excluded from the study. In patients treated with the combination of silver foam dressing with Zn-hyaluronic gel, the dressing was checked on the 2nd day and removed on the 6th or the 7th day. Considering the fact that our method is a special combination of the dressing, an ethical approval was not requested by the Ethical Committee of our institutions for this prospective study. ∗Institutional review board: Clinical application of the combination of the dressing has been accepted and permitted in 2010 by our medical board of the Hungarian Pediatric Surgery Committee. Possible benefits and complications, along with other treatment options, were explained to the parents of each child.

## 3 Results

**Figure 1:**
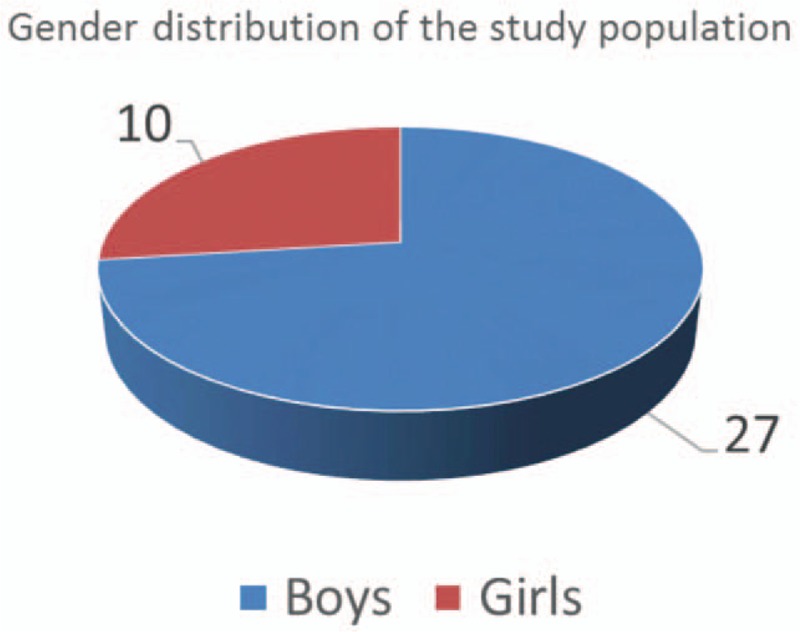
Gender distribution of the study population.

Most of the studied children were <5 years of age. With regards to gender 138 distribution, out of the 37 injured children, 27 were boys and 10 were girls. This gender ratio 139 is similar to international and European incidence rates, namely that boys (73%) are more likely 140 to be exposed to burn injury (Fig. 1).

**Figure 2:**
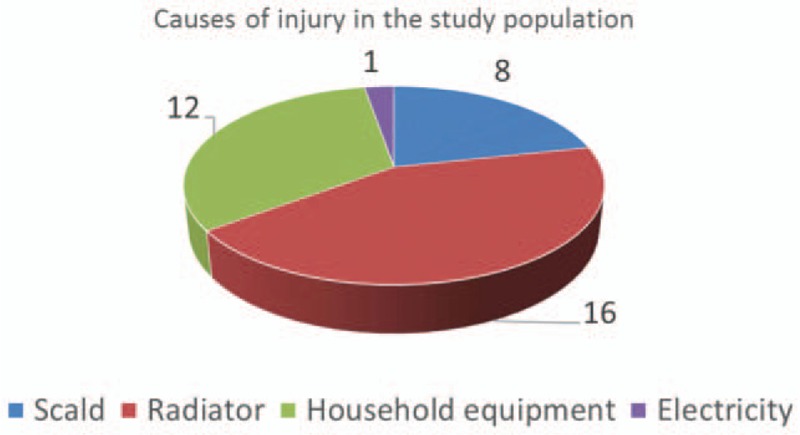
Causes of injury in the study population.

Concerning the causes of the hand burns, touching a heater or a stove with the palm of the hand was the most common (16/37 pts), while injuries caused by household equipment (12/37 pts) and hot water (8/37 pts) were also frequent. Only 1 child in the sample had injuries caused by electricity (Fig. 2).

In patients treated with the Zn-hyaluronan gel combined with Aquacel Ag foam dressing, no wound infection was diagnosed, corresponding to an infection ratio of 0/37. The bandages were kept in place and usually uncontaminated for the duration of the treatment.

**Figure 3:**
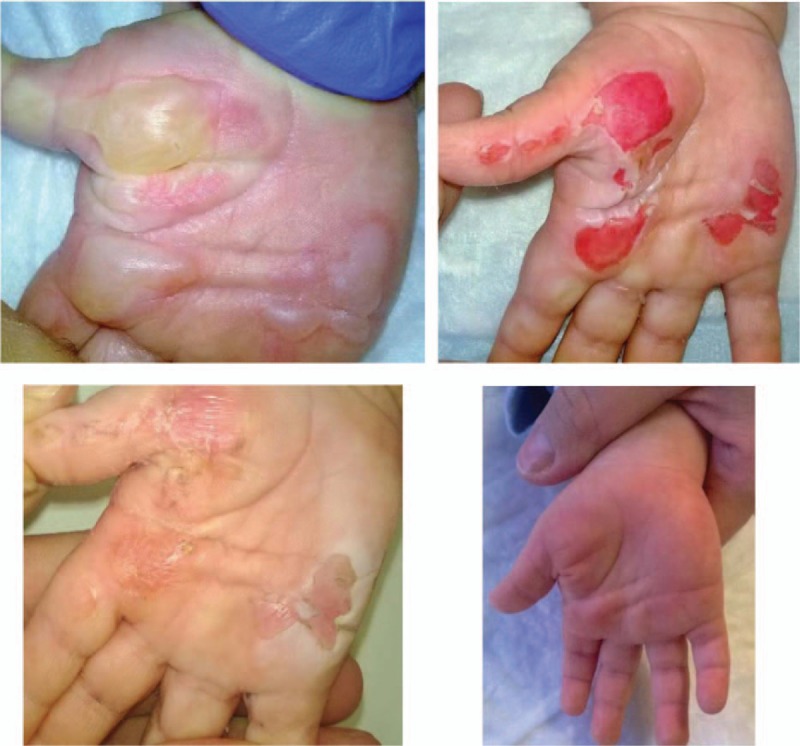
Superficial second degree burn injury of the right hand before (A), and after the removal of bullae (B). On the 7th day after initiation of the treatment, the burn injury is healed (C). One month after the injury, the cosmetic result is good (D).

In general, epithelialization of the burned area was observed 6 to 7 days after primary treatment (Fig. 3 A-D), which corresponds well with the results from other methods of dressing for this type of burn. In our recent study, the same combined treatment resulted in similarly improved outcome when we applied it to partial thickness burn injuries of body parts other than the hands in children.^[9]^

## 4 Discussion

Burn injuries often occur in families with lower social and economic status, where the danger of an infection during the healing process is also higher. It is important that, where possible, childhood burns should be treated in a pediatric surgical department or in a burn centre. We conducted a prospective study for 4 years in 1 centre in Hungary, in which we treated 37 children with superficial and mixed-typed second-degree hand burns with Aquacel Ag foam and Zn-hyaluronan gel applied simultaneously. Children with deep second-degree hand burns (II/B or II/2) were excluded from the study.

The limitations of our study are that it was conducted in 1 centre only and involved only 1 method. The patients were not control-matched and not randomized. Because of the study design, we did not include children with II/B degree burn depth in this investigation; whether the same combined treatment would be beneficial in such cases too remains a subject for future studies.

All the patients diagnosed with a burn injury <5% TBSA were treated with this method. Because of the modern dressings, epithelialization generally occurred on the 6th day, as in a previous study by an independent group.^[1]^ According to our results, in our experience there were no cases of infection.

Conservative treatment of hand burns with the widely used local remedy, silver-sulfadiazine ointment, creates a heavy, oozing fatty layer that is difficult to tolerate. This thick, adherent layer also makes proper determination of burn depth very difficult. Prior to this study, the silver-sulfadiazine was the gold standard for the treatment of superficial burns in our centre. Disadvantages of that treatment consisted of the need for daily dressing changes and difficulties with assessing burn depth.^[4]^

In the children treated with traditional methods, anesthesia had to be used every day or every other day to change the dressing, while the foam dressing containing silver could be used until the wound healed, therefore repeated anesthesia was not required.

Treatment of mixed-type burns is still a big challenge, and whether conservative treatment is sufficiently effective or not has remained a widely discussed topic. Treatment for a coherent and deep second-degree burn wound is a surgical intervention, whereas mixed-type second-degree burns can also be effectively treated with conservative methods.

In nearly 90% of the children, we used Aquacel Ag foam dressing with Curiosa (Zn-hyaluronan) gel at the first intervention. We checked the dressing on the 2nd day and removed it on the 6th or 7th day. Second day control was important to check the dressing condition. If the bandage was clean, then changing of the dressing was not necessary. In those cases, when we found that the dressing was contaminated, we changed it, which explains the rational of why we checked the wound on the 2nd day.

Hydrofiber dressing containing silver combined with hyaluronan gel containing zinc tends to be effective against infections and promotes wound healing. The dressing is comfortable and can be applied easily to the hand. It also creates an appropriate environment for proper wound healing.^[6,8]^ In contrast to traditional treatments, applying, changing, and removing a combination of Aquacel Ag foam with Zn-hyaluronan dressing is painless. A very important aspect of this new method is that the physical strain and stress of the child is reduced because of fewer control check-ups and dressing changes. On average, 2.5 dressings were used per case. Because of the reduced number of dressings and anesthesia required, the approximate cost of the treatment per child was cut by half. Currently, there are only a few clinical studies available in the literature about the application of Aquacel Ag foam dressing in pediatric patients with partial thickness burns.^[10–13]^ In these studies, length of hospital stay was significantly shorter in the Aquacel Ag group.^[10,13]^ Moreover, dressing frequency was 3 to 4 times lower in the Aquacel Ag group than in the standard dressing group.^[8]^

## 5 Conclusion

According to our results in the present study, Aquacel Ag foam dressing combined with Zn-hyaluronan gel can be considered as an effective, gentle, and child-friendly treatment, which ensures preferential wound healing and subservient cosmetic results. It has to be noted, however, that this dressing should be applied only after an exact assessment of the burn's depth.

## Author contributions

**Conceptualization:** G. Jozsa.

**Formal analysis:** A. Garami.

**Supervision:** Z. Juhasz.

**Validation:** P. Vajda.

**Writing – original draft:** A. Csenkey.

**Writing – review & editing:** G. Jozsa.
